# Finite element analysis of stress concentration in Class V restorations of four groups of restorative materials in mandibular premolar

**DOI:** 10.4103/0972-0707.45251

**Published:** 2008

**Authors:** Shubhashini N, Meena N, Ashish Shetty, Anitha Kumari, Naveen DN

**Affiliations:** Department of Conservative Dentistry and Endodontics, V. S. Dental College and Hospital, Bangalore, Karnataka, India

**Keywords:** Class V, FEM (Finite Element Model), finite element study, stress concentration

## Abstract

**Aim:**

To study the concentration of stress in class V restoration of four different restorative materials subjected to occlusal load of 100N, 150N, 200N, 250N and to analyse the obtained data with the listed properties of the restorative material.

**Materials and Methods:**

Using FEM analysis the stresses generated in a class V lesion in a mandibular premolar was studied.

**Results:**

Within the framework of the aforementioned views, and from the results of the study it can be concluded that microfilled composite is the most suitable restorative material followed by flowable composite, glass ionomer cement and resin modified glass ionomer cement.

**Conclusion:**

Restoration of Class V lesions with materials of higher modulus of elasticity will enable better stress distribution.

## INTRODUCTION

The human tooth is a marvel of nature. However, it has one significant shortcoming; it has only a limited capacity for regeneration. This necessitates the replacement of tooth structure lost as a result of caries, trauma or other reasons, with a suitable restorative material.

The restoration of most surfaces of tooth is a fairly successful procedure. This is because of the improved understanding of caries and other disease processes, as well as due to the advancements in restorative materials and techniques. However, restoration of cervical lesions – both carious and noncarious defects – presents a unique clinical situation, because the etiology of such lesions is not always clear. The teeth are subjected to occlusal loads constantly. Besides, other factors like location of the tooth, abnormal occlusal stresses, age of the patient and the restorative material used have a significant influence on the retention rates.[1]

Traditionally, restorative treatment of cervical lesions was carried out by preparing Class V cavities and restoring them with various materials like silver amalgam, gold, porcelain, silicates etc. All these materials have some disadvantages and generally require removal of moderate amounts of the remaining tooth structure. With the advent of adhesive restorative materials, the need for preparing a cavity has long been overcome and simple conservative treatments, which utilize bonding for retention, have become more popular. Materials having adhesive potential are composites, polyacid modified glass ionomer, glass ionomer, resin modified glass ionomer and a combination of glass ionomer and composite in a bi-layered technique.[1] Various methodologies have been used to study the stress concentrations in the cervical region, namely, articulated study models, photo elastic studies, strain gauge studies.

Finite element analysis (FEA) is one of the more recently used techniques for stress analysis. The basic concept of this technique is the visualization of actual structure as an assemblage of a finite number of elements. Finite element analysis divides the problem domain into a collection of smaller parts (elements). An overall approximated solution to the original problem is determined.

Various software such as NASTRAN, Auto FEA, IDEAS, IANA, IBM Finite element modeler, LS Dyna, NISA II are available for FEA.

## MATERIALS AND METHODS

A three-dimensional mathematical finite element analysis model was generated for analysis, using an intact normal extracted human mandibular second premolar. The mandibular second premolar was selected for the study, as the incidence of cervical lesion is most common on the buccal aspect of lower premolars.[1]

### Modeling of a normal lower premolar tooth

**Figure 1:**
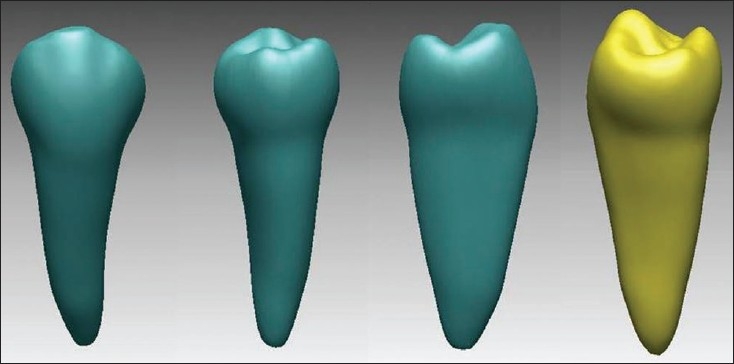
Model of mandibular premolar

The first step in a finite element analysis is modeling. The quality of the analysis results depends on the accuracy of the model. The tooth was subjected to a CT (computerized tomography) scan and the cross section of the tooth was obtained at an equal interval of 0.5 mm. In each cross section, the different layers of the tooth were viewed accurately. [Figure 1]

These sections were obtained in DICOM format and the data was fed to the computer. [DICOM (Digital Imaging and Communication of medicine) is a neutral image format basically for medical imaging purposes like CT, MRI (magnetic resonance imaging etc.]

Using the software MIMICS, these cross sections were converted into a three-dimensional model. [Materialise's Interactive Medical Image Control System (MIMICS) is an interactive tool for the visualization and segmentation of CT images, as well as MRI images and 3D rendering of objects.] Thus a virtual model of the second mandibular premolar was obtained.

Bone was created as a rectangular block. The root portion was inserted inside the bone. This bone was constrained for motion in all the directions. The model was exported to NASTRAN.

### Meshing

**Figure 2:**
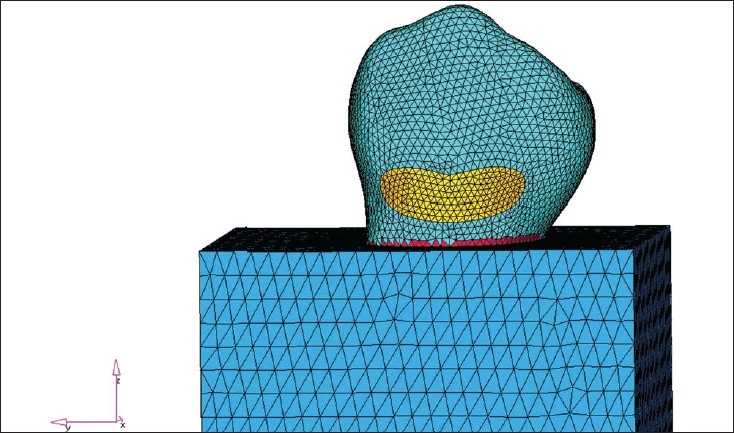
Model after meshing

The creation of the Finite Element Model (FEM) was the next step. The Finite Element Model was divided into several Finite Elements. The element chosen for the study was C Tetra, which is a 4-noded element. NASTRAN was used to create the Finite Element Model. [Figure 2]

### Dimensions of the virtual cavity

**Figure 3:**
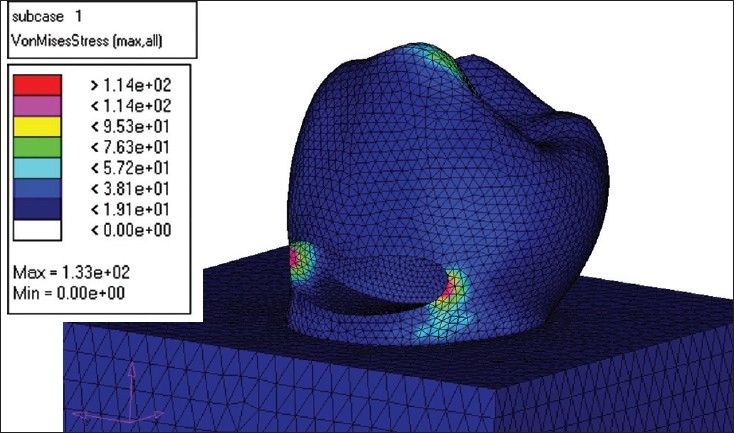
Model demonstrating stress without restoration

Class V cavity measuring 2 mm gingivo-occlusally, 3 mm mesiodistally and 1.5 mm in depth was held constant, with occlusal margin in enamel and gingival margin in dentine. The internal line angles of the cavity were rounded, in order to prevent any stress concentration.[2] [Figure 3]

### Loads and constraints

Following the meshing of the tooth model and the bone block, the outer element of the bone was fully constrained. The generated model, unrestored and restored with the above mentioned materials, was then subjected to an occlusal load of 100N, 150N, 200N and 250N, at right angles to the buccal cusp at a point 0.4 mm inside the buccal cusp tip, to simulate the effect of tooth contact in a lateral excursive movement.

The load was applied and the stress analysis was carried out using the NASTRAN solver. The stress distributions have been plotted using the general post processor of NASTRAN.

### Restoration of the lesion

**Figure 4:**
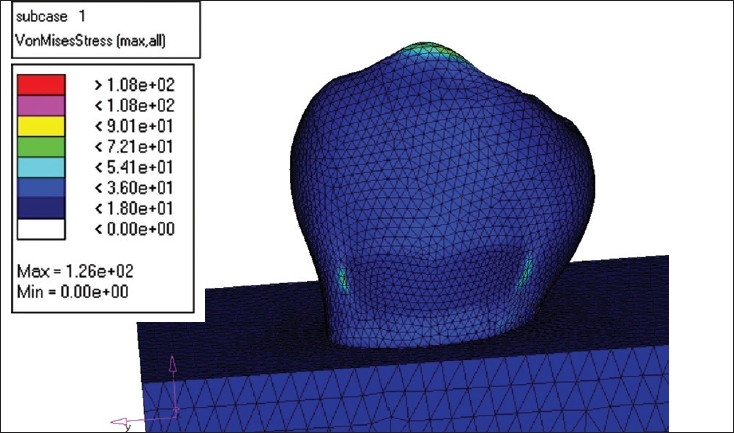
Flowable composite load -250 N

**Figure 5:**
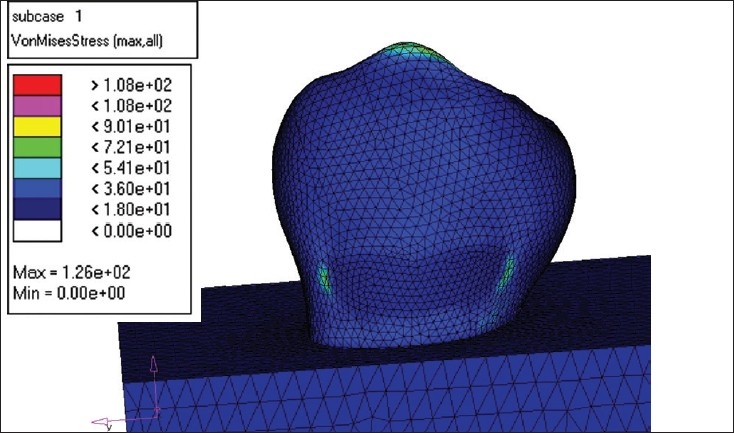
Glass ionomer load 250 N

**Figure 6:**
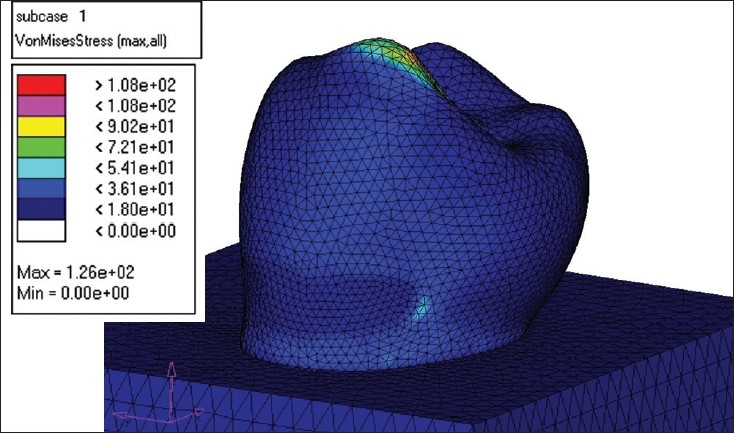
Microfilled composite load -250N

**Figure 7:**
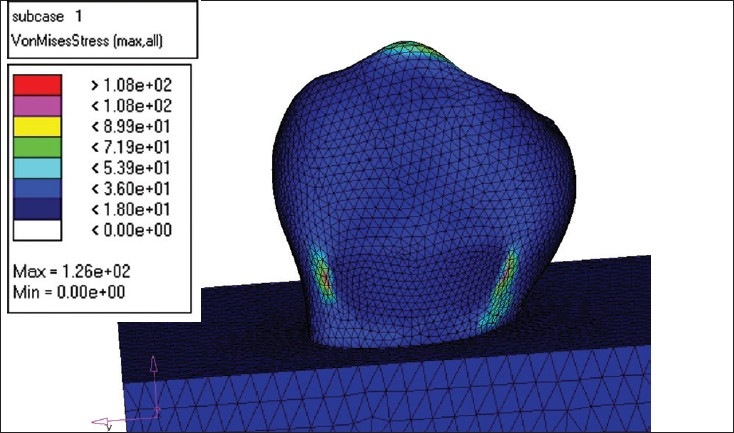
Resin modified GIC load- 250 N

- MODEL I - This model was restored with microfilled composite (Silux plus) [Figure 4].
- MODEL II - The second model was restored with flowable composite (Tetric Flow) [Figure 5].
- MODEL III - The third model was restored completely with glass ionomer cement (Ketac Bond) [Figure 6].
- MODEL IV - The fourth model was restored with resin modified glass ionomer cement (Vitrebond) [Figure 7].

**Figure 8:**
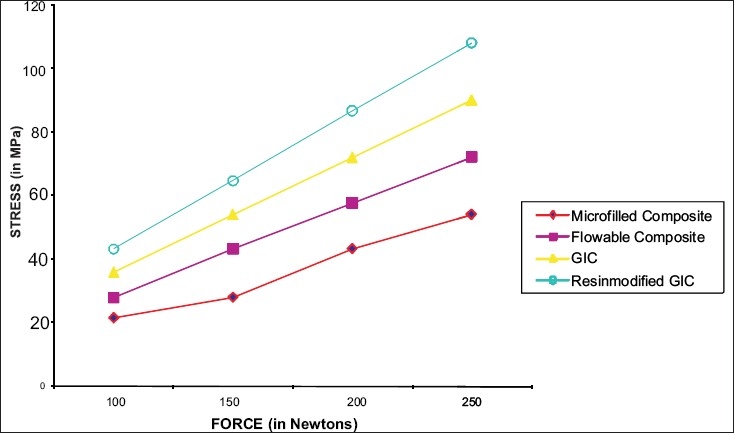
A comparison of the stress values in all the four restoration groups under different occlusal loads

Stress analysis was performed with 100N, 150N, 200N, and 250N loads, using the NASTRAN solver.

## RESULTS

The principal stresses in each of the models were studied. The results are presented in terms of the Von Misses stress values. As the tensile strength values of all the concerned materials were not available for comparison, the likelihood of failure is decided on the basis of Von Misses stress value [Table 1].[3]

**Table 1:** Stress values of the restoration groups at different loads

Applied load in Newton	100	150	200	250
Without restoration	21.6 MPa	84.6 MPa	112.8 MPa	141 MPa
Microfilled composite (Silux plus)	21.6MPa	28.1MPa	43.3 MPa	54.1 MPa
Flowable composite (Tetric Flow)	28.0 MPa	43.3 MPa	57.7 MPa	72.1 MPa
Glass ionomer cement (Ketac Bond)	36.0 MPa	54.1 MPa	72.1 MPa	90.1 MPa
Resin modified glass ionomer cement (Vitrebond)	43.2 MPa	64.7 MPa	86.7 MPa	108.0 MPa

## DISCUSSION

Cervical lesions are common and they pose a challenge to the dental profession. It is likely that their prevalence will increase, as the world's population ages. The location of these lesions makes it difficult to provide a long-lasting restoration. The prevalence of noncarious Class V lesions has been estimated to be between 31 and 56%. It has also been estimated that 85% of the population shows some loss of tooth structure at the cervical area.[4]

Over the last decade or so, a biomechanical theory (tooth flexure theory) has been postulated, suggesting that mechanical overloading of cervical enamel caused by cuspal flexure may contribute to noncarious cervical tooth loss. As the tooth flexes, the cusps are subjected to an axial compression load, resulting in cervical tensile and shear stresses acting at right angles to an axial load in a manner similar to a diametral compression test. This results in breakdown of the bonds between the hydroxyapatite crystals, which leads to crack formation, which eventually results in enamel loss. This mechanism has been termed ‘abfraction’.[5]

Abfraction lesions are wedge-shaped defects that are principally found on the buccal and labial aspects of the teeth. There seems to be a strong association between abfraction lesions and parafunctional habits. Previous studies have found that stresses concentrate in the thin cervical enamel area and the magnitude of these stresses exceeds the known failure stresses for enamel.[6]

A higher frequency of cervical lesions has been noted in mandibular teeth than in maxillary teeth. In recent studies, retentive failure rates of restorations were also found to be higher in the mandibular arch than the maxillary arch. The higher incidence and increased failure rate of restorations are consistent with the lingual orientation of the mandibular teeth. It renders them more susceptible to the concentration of tensile stresses at the cervical cross section of the mandibular teeth, particularly the bicuspids and may also contribute to the failure of these teeth to withstand tensile stress. The difficulty in moisture control of mandibular teeth during the restorative process and less number of open tubules in mandibular dentin have also been mentioned as possible contributory factors to the increased failure rate.[1]

Numerous clinical trials have been undertaken involving Class V restorations, but no consensus currently exists regarding the long-term clinical performance of any restorative material. Many questions regarding material selection and clinical techniques are still unanswered.

Treatment of Class V lesions presents a unique challenge. Leakage and lost restoration are a common observation among clinicians. Tooth location, stressful, occlusion, type of restorative material and the age of the patient have significant influence on the retentive failure rates.[7]

Resin composites and glass ionomer cements are widely used for restoring cervical lesions, as they are esthetic and mercury free, and they bond to the tooth structure. One of the main problems that clinicians face when restoring Class V or cervical cavities with composite resin is dealing with the problems of polymerization shrinkage. The shrinkage which accompanies the setting of these materials results in marginal gap formation in the order of 10-15*µ*m and this gap often remains open, despite swelling of the restoration following water absorption. These gaps between the tooth margin and restoration lead to clinical problems such as post-operative sensitivity, stained margins and recurrent caries.[8]

Microfilled resins tend to flex with the tooth rather than debond, as compared to the more rigid macrofilled resin restorations.[8] A thickness of resin of up to 80µm would be helpful in increasing the strain capacity of Class V composite restoration, so that it will be better able to resist the forces generated by polymerization shrinkage.[7]

Various methodologies have been used to study the stress concentrations in the cervical regions. These include Articulated study models,[9] Photo elastic studies,[10] Strain Gauge studies[11] and Finite element study.[12] The advantages of FEA are numerous and important. A new design concept may be modeled to determine its real world behavior under various load environments. It has the ability to obtain accurately the stress pattern throughout the structure under consideration, even if the structures to be analyzed are non homogenous. Once a detailed computer aided design model has been developed, FEA can analyze the design in detail, saving time and money by reducing the number of prototypes required. At present, improved computers, modeling techniques, affordable computer workstations and professional assistance render the finite element method a very reliable and accurate method in biomechanical applications.

In this method, solutions for each element are combined to obtain a solution to the body. Among the various methods for assessing deformations produced in different structures, FEA has proven its efficiency in many ways, from the normal situations concerning the nature of tooth movement under orthodontic loads to special situations like alveolar bone loss, use of extra oral forces systems in orthodontics etc. The other experimental methods may indicate the ultimate load for failure of a tooth, but may not provide satisfactory answers regarding the development mechanism of such a failure. However, with FEM, the intermediate levels of a process can easily be understood and it is most suitable for the modeling of an asymmetrical tooth structure.

This study investigated the strength properties of various resin based restorative materials recommended for use in Class V applications.[7] The purpose of this study was to evaluate the mechanical behavior of the materials under different loading. The possible changes in tooth structure because of the presence of cavity and the utilized restorative material and their consequences were studied in detail. The study sheds light on safe utilization of restorative materials and strictly considers only the mechanical aspects, as it is an *in vitro* virtual model study.

Using finite element analysis, stresses generated in a Class V lesion unrestored and restored with various groups of materials were evaluated. A mandibular premolar was used in the study, as a higher frequency of cervical lesions has been noted in mandibular teeth. The cavities that destroy the structural integrity of an intact tooth leads to the development of stress concentration near cavity vicinities (in the mesio-axial and disto-axial proximities).[3]

In a restored tooth, when the loading angle and the restorations size were kept fixed, increasing the load was found to increase the Von Misses stress in the immediate vicinity of the restored area and it was found to be inversely proportional to the Young's modulus value of the restorative material. As the value of Young's moduli of the enamel and the restoration material do not match, it inevitably disrupts the otherwise continuous structure and gives rise to stress concentrations. If the restoration material has larger Young's modulus, the destruction becomes less prominent. The increase in the load does not cause a change in the overall stress pattern, but only shifts the value to a higher scale. In all the models considered, the Von Misses developed in the unrestored tooth was found to reach higher values than the stresses developed in the restored model.[3]

Applying this data to a real model, one can suggest that the loading at which the tooth is being subjected to may not create immediate failure in the tooth, but may create cracks in the tooth structure; over a period of time, it may lead to a complete failure. As the formation of non-carious lesions/cavities are an inevitable part of various etiological factors, their possible negative effects should be foreseen and necessary precautions should be taken. From the mechanical point of view, restoration of these defects is important and the best clinical approach would be to apply restorative materials, which have large Young's moduli.[3,13] This is in accordance with the conclusion drawn by Yaman, Sahin and Adyin, in their study.

One can draw a similar conclusion from the present study, as microfilled composite (9.5GPa) performed best with a stress value of 54.1MPa at a load of 250N, followed by flowable composite (6.2GPa)[14] with a stress value of 72.1MPa, glass ionomer cement (4.35GPa) with a stress value of 90.1 MPa and resin modified glass ionomer cement (1.1GPa) with a stress value of 108MPa.

A virtual model cannot completely mimic a real biological model, i.e. a set of teeth cushioned by periodontal ligament and perpetually being subjected to various types of loading stresses. Therefore, any conclusion drawn by considering a single stress component will not reflect the true behavior of the tooth and hence will be far from accurate. Similarly, as the tooth represents a highly irregular structure, any two-dimensional analysis will fail to represent the actual tooth. In addition, FEA itself has its own limitations such as - solving a large number of complex equations consume a lot of time; the accuracy is limited to the software and hardware capabilities of the computer; and, it is governed by the element inputs. Wrong inputs can lead to design deficiency.

In spite of these limitations, FEM is one of the most widely used stress analysis in dentistry today, as it is most suitable for modeling an asymmetrical structure like the tooth. Finite Element Analysis is a method wherein one can visualize and study the stresses generated in a tooth, restoration, restoration-tooth interface etc., simultaneously for different occlusal/incidental forces, thus generating a virtual picture of biomechanical characteristics of any restoration. This helps us in predicting the probable success of a restoration for a given clinical situation. Improved computers and modeling techniques render the FEM a very reliable and accurate estimation approach in biomechanical applications.

## CONCLUSION

This study using finite element analysis evaluated stresses generated in a Class V lesion unrestored and restored with glass ionomer, resin-modified glass ionomer, microfilled composite, flowable composite and subjected to load of 100N, 150N, 200N, 250N. A mandibular premolar was used in the study and the principal stresses developed during various occlusal loads were evaluated.

The results of the study suggest that –

- The cavities that destroy the structural integrity of an intact tooth lead to the development of stress concentrations near the cavity vicinities.
- The loading at which the tooth is being subjected to may not create immediate failure in the tooth, but may create cracks in the tooth structure. The applied load may eventually lead to a complete failure.

The following observations were drawn from the analysis:

- When a tensile load is applied on a tooth with Class V lesion, there is a high stress concentration in the mesio-axial and disto-axial proximities.
- Restoration of these defects helps in the reduction of stress concentration.
- The stress concentration is in the immediate vicinity of the restored area and was found to be inversely proportional to the Young's modulus value of the restorative material used.
- From the biomechanical point of view, it can be said that the best approach is to apply restorative materials which have as large as possible Young's moduli.

Within the framework of the aforementioned views, it can be concluded that for Class V application, Microfilled composite performed the best, followed by Flowable composite, Glass Ionomer cement and Resin modified glass ionomer cement.
